# Emerging Cancer Immunotherapies: Cutting-Edge Advances and Innovations in Development

**DOI:** 10.3390/medsci12030043

**Published:** 2024-08-28

**Authors:** Monica Maccagno, Marta Tapparo, Gabriele Saccu, Letizia Rumiano, Sharad Kholia, Lorenzo Silengo, Maria Beatriz Herrera Sanchez

**Affiliations:** 1 Department of Molecular Biotechnology and Health Sciences, 10126 Turin, Italy; letizia.rumiano@unito.it; 2 Molecular Biotechnology Centre, University of Torino, 10126 Turin, Italy; marta.tapparo@unito.it (M.T.); gabriele.saccu@unito.it (G.S.); sharadkumar.kholia@unito.it (S.K.); lorenzo.silengo@unito.it (L.S.); 3 Department of Medical Sciences, University of Torino, 10126 Turin, Italy; 4 2i3T, Società per la Gestione dell’incubatore di Imprese e per il Trasferimento Tecnologico, University of Torino, 10126 Turin, Italy

**Keywords:** immunotherapy, cell therapy, cancer, CAR-T, CRISPR/Cas9, LNPs, vaccine

## Abstract

The rise in biological therapies has revolutionized oncology, with immunotherapy leading the charge through breakthroughs such as CAR-T cell therapy for melanoma and B-ALL. Modified bispecific antibodies and CAR-T cells are being developed to enhance their effectiveness further. However, CAR-T cell therapy currently relies on a costly ex vivo manufacturing process, necessitating alternative strategies to overcome this bottleneck. Targeted in vivo viral transduction offers a promising avenue but remains under-optimized. Additionally, novel approaches are emerging, such as in vivo vaccine boosting of CAR-T cells to strengthen the immune response against tumors, and dendritic cell-based vaccines are under investigation. Beyond CAR-T cells, mRNA therapeutics represent another promising avenue. Targeted delivery of DNA/RNA using lipid nanoparticles (LNPs) shows potential, as LNPs can be directed to T cells. Moreover, CRISPR editing has demonstrated the ability to precisely edit the genome, enhancing the effector function and persistence of synthetic T cells. Enveloped delivery vehicles packaging Cas9 directed to modified T cells offer a virus-free method for safe and effective molecule release. While this platform still relies on ex vivo transduction, using cells from healthy donors or induced pluripotent stem cells can reduce costs, simplify manufacturing, and expand treatment to patients with low-quality T cells. The use of allogeneic CAR-T cells in cancer has gained attraction for its potential to lower costs and broaden accessibility. This review emphasizes critical strategies for improving the selectivity and efficacy of immunotherapies, paving the way for a more targeted and successful fight against cancer.

## 1. Introduction

Despite advancements in early detection methods and therapies, cancer remains a significant global health challenge with high incidence and mortality rates. According to the World Health Organization, cancer caused approximately 10 million deaths in 2022, accounting for one in six deaths globally [1]. Common cancers among women include breast, lung, colorectal, thyroid, and cervical cancers, while prostate, colorectal, lung, liver, and stomach cancers are prevalent among men [2]. Even though death rates have been declining in several cancer types due to effective treatments, much of cancer research is dedicated to developing improved therapies to further reduce mortality rates.

Immunotherapy has emerged as an advanced treatment approach for a range of cancers, encompassing both hematological malignancies and solid tumors. This strategy harnesses the patient’s immune system to combat cancer, offering a pathway to more targeted and efficient treatments. Compared to chemotherapy, immunotherapy is associated with fewer side effects, making it a promising therapeutic option for various types of cancer [3].

The idea of using the immune system to fight cancer has been around since the 18th century, but it gained renewed attention and significant progress in the 20th century due to new technological advancements. In the 1900s, Paul Ehrlich, Lewis Thomas, and Sir Frank Macfarlane Burnet independently suggested that the immune system has the ability to detect and attack cancer cells through tumor-associated antigens, similar to how it rejects foreign tissue grafts [4]. Support for this hypothesis grew with evidence of successful immune responses in mice after tumor transplants and clinical reports of melanoma regression in patients with autoimmune diseases. However, a comprehensive understanding of the underlying mechanism was lacking until the advent of knockout mouse models, which provided experimental proof linking immunodeficiency to cancer [5]. Advances in molecular and biochemical research later identified tumor-specific immune responses, especially those involving T cells, confirming the immune system’s ability to fight cancer. However, tumors have developed various strategies to evade immune surveillance, including impairing the antigen presentation machinery, enhancing negative immune regulatory pathways, and recruiting immune cells that promote tumor growth [6,7]. These immunosuppressive strategies block the function of anti-tumor immune cells, making it challenging to sustain effective anti-tumor immune responses. Despite heterogeneity between cancer types and populations, the role of the tumor microenvironment (TME) in tumor progression remains consistent [8]. Immunotherapy aims to restore the ability of anti-tumor immune cells, especially cytotoxic T lymphocytes (CTL), to destroy tumors. However, the presence of pro-tumor immune cells such as tumor-associated macrophages (TAMs), regulatory T cells (Tregs), myeloid-derived suppressor cells (MDSCs), and group 2 innate lymphoid cells (ILC2s) significantly impair anti-tumor immune responses and create an immunosuppressive TME [8]. 

Based on our understanding of tumor evasion and an immunosuppressive TME, several cancer immunotherapies have been developed to alter the TME and combat tumor cells. For instance, inhibiting immune checkpoints like PD-1/PD-L1 and CTLA-4 can alleviate the functional inhibition of T cells [9]. Transforming immunosuppressive M2-type TAMs into pro-inflammatory M1-type (dual blocking of PI3K-γ pathway and CSF-1/CSF-1R) can reduce immunosuppression and enhance CD8^+^ T cell activation [10]. Dendritic cell (DC)-based vaccines can stimulate T cell responses by overcoming antigen presentation inhibition [11]. Chimeric Antigen Receptor (CAR) cell therapy, which involves genetically engineering the patient’s T cells to express specific receptors that can target and eliminate cancer cells [12], amongst others. 

Despite the success of some immunotherapy approaches, several factors limit the activation of tumor-specific immune responses. These include mechanisms of resistance involving T cell immune checkpoint pathways, intratumoral heterogeneity, inadequate production and function of tumor-specific CD8^+^ T cells, and scarcity of suitable neoantigens with impaired processing and presentation [13]. Hence, the discovery of new immune checkpoints and exploring novel molecular pathways are critical to overcoming immune evasion. 

**Figure 1:**
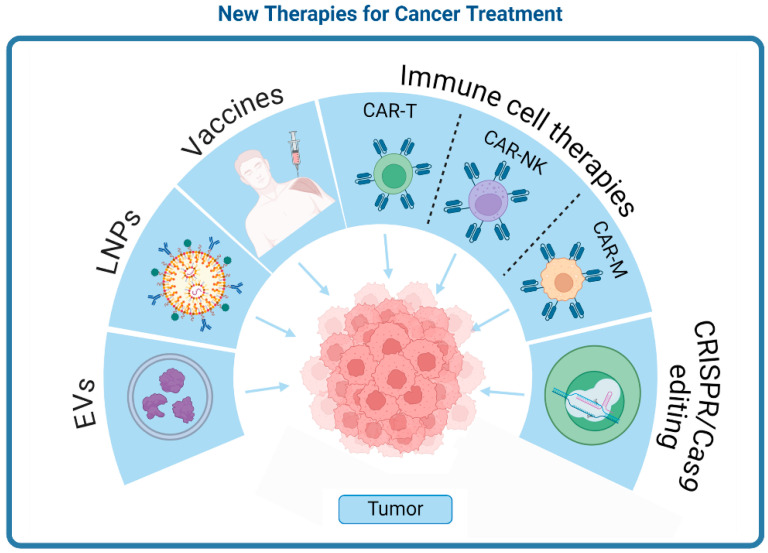
Graphical representation of new therapies and approaches in the anti-tumor field.

In this review, we explore several novel approaches that are emerging in the field of immunotherapy (Figure 1). These include in vivo vaccine boosting of CAR-T cells to enhance the immune response against tumors and the investigation of dendritic cell-based vaccines. The review also examines the potential of T cell-targeted delivery of mRNA using lipid nanoparticles (LNPs). Additionally, we discuss how CRISPR/Cas9 technology has effectively enhanced the proliferation and persistence of CAR-T cells by creating a memory phenotype, reducing exhaustion, and identifying new targets, thus improving their antitumor potential [14]. Extracellular vesicles (EVs) have emerged as vital players in intercellular communication, influencing various cellular processes by carrying proteins, lipids, and nucleic acids. The review explores how EVs from both tumor and immune cells can modulate immune responses and the tumor microenvironment, as well as how integrating EV functionalities into CAR-T therapy and adopting CAR-T cell-derived EVs offer a novel approach to enhance therapeutic efficacy and address current limitations [15]. We also cover banking donor or stem cell-derived T cells for off-the-shelf CAR-T cell therapy, which, despite relying on ex vivo transduction, can be cost-effective, simplify manufacturing, and expand treatment to patients with low-quality T cells [16]. Overall, this review emphasizes critical strategies developed or currently in development aimed at enhancing the selectivity and efficacy of immunotherapies, paving the way for more targeted and successful cancer treatments.

## 2. Immune Cell Therapy

Adoptive cell therapy (ACT) using autologous peripheral lymphocytes or tumor-infiltrating lymphocytes (TILs) after stimulation and expansion by lymphokines has significantly advanced cancer treatment and immunotherapy in recent decades. Significant clinical effects have been shown only in certain tumors, such as melanoma. Advances in gene engineering have accelerated the development of CAR-T. These are T cells extracted from a patient’s own body and then genetically modified to specifically identify and destroy cancer cells. Compared to ACT, CAR-T therapy represents a more sensitive and precise approach [17].

**Figure 2:**
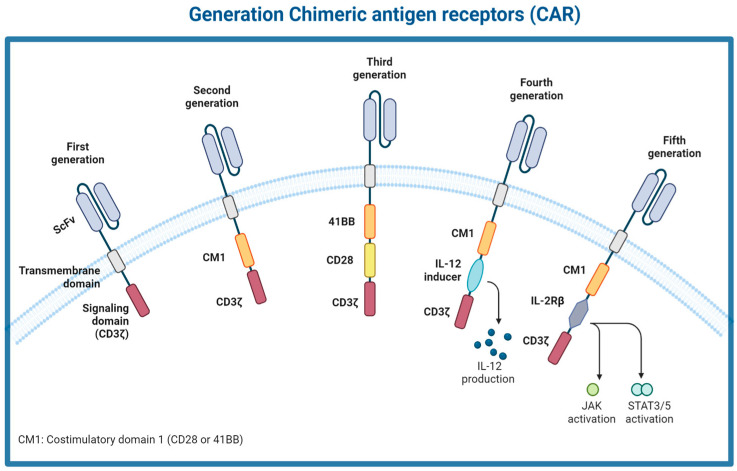
Five generations of CARs. The 1st generation of CAR consisted of an extracellular single-chain variable (scFv) and an intracellular CD3ζ activation domain. Building on this, the 2nd generation of CARs incorporated an additional co-stimulation domain. The 3rd generation advanced further by integrating two co-stimulation domains. The 4th generation, also known as TRUCKs, included a cytokine inducer domain to enhance cytokine-mediated cytotoxicity. The 5th generation introduced an IL-2Rbeta domain, which activates the cytokine receptor-dependent JAK/STAT pathway.

Over the years, several improvements have been made across the generations of CAR constructs. The first generation of CARs contained a single-chain variable fragment (scFv) and a single CD3ζ subunit, which is the intracellular component of the CD3 complex that mediates MHC-TCR signaling transduction to activate T cells. Due to the poor efficacy of the first-generation CAR-T cells, a co-stimulatory signaling domain (CD28 or 4-1BB) was added in the second generation to amplify the signal. The third generation of CARs combined both CD28 and 4-1BB, providing T cells with stronger activation signals. Recently, fourth- and fifth-generation CARs have been developed. The fourth generation, also known as TRUCKs (T cells Redirected for Universal Cytokine Killing), included one or more co-stimulatory signaling domains and inducible or constitutive expression of pro-inflammatory cytokines (e.g., IL-12, IL-10) [18]. The fifth generation incorporated a cytokine receptor domain that activates a cytokine signaling cascade, further enhancing T cell activation and function [19] (Figure 2).

To date, the FDA has approved six CAR-T therapies (Supplementary Table S1) [20,21,22]. Notably, CAR-T therapy has proven particularly effective in treating blood cancers. However, solid tumors represent 90% of human cancer, and CAR-T has been shown to be less effective in solid tumors due to major challenges: immunosuppressive TME and lack of tumor-exclusive targets [23]. Thanks to the new generation of CAR-T armored by expressing cytokines and modified by CRISPR/Cas9 technology to delete negative regulators of T cells to enhance antitumor activity, several clinical trials have started targeting solid tumors [24].

Beyond CAR-T, since 2010, the development of armored CAR-T and TCR-T (T cell Receptor T cell) therapies has expanded the scope of genetically engineered T cell treatments for cancer. Autologous or allogeneic TCR-T cells can be engineered to express TCRs that recognize multiple combinations of specific peptides and human leukocyte antigens (HLA) through advancements in TCR isolation, sequencing, and genetic engineering techniques. Unlike CAR-T cells, which target antigens on the cell surface, TCR-T cells can recognize a broader range of tumor-associated antigens, including those derived from intracellular proteins [25]. However, TCRs cannot mediate signal transduction on their own due to their short cytoplasmic tails and require CD3 for signal transduction [26]. The advantages of TCR-T therapy include its high specificity for cancer cells, its ability to target a wider range of antigens, including intracellular ones, and its increased potential in treating solid tumors. TCR-T therapy is still largely in the experimental and clinical trial stages, but it has shown promise in treating various cancers, including melanoma, synovial sarcoma, and certain types of gastrointestinal cancers [27,28].

Several factors contribute to the failure of CAR-T cell therapy, including patient disease progression, insufficient T cell harvest, delays in CAR cell manufacturing, low CAR cell expansion and persistence, intrinsic T cell defects, antigen escape, and systemic cytotoxicity (e.g., cytokine release syndrome or neurotoxicity). To address these challenges, various approaches have been explored to enhance CAR-T cell efficacy. These include potentiating T cell metabolism and combinatorial therapies with small molecule inhibitors. One strategy involves culturing CAR-T cells in a medium supplemented with linoleic acid (LA), which shifts CAR-T cell metabolism, reduces exhaustion markers, increases the memory phenotype, and improves the tumor-killing capacity of both mouse CD8 and human CAR-T cells [29]. Additionally, Bergaggio et al. used lorlatinib, an Anaplastic Lymphoma Kinase (ALK) inhibitor, to boost ALK receptor expression in neuroblastoma tumors, thereby enhancing the effectiveness of ALK.CAR-T therapy [30]. Furthermore, researchers have engineered Natural Killer (NK) cells and macrophages to create CAR-NK and CAR-M cells as alternative methods for adoptive cell therapy [31]. 

NK cells are cytotoxic lymphocytes of the innate immune system that can quickly respond to non-self cells. Unlike T cells, which recognize antigens presented on MHC molecules, NK cells can directly recognize and target cells without the need for MHC. CAR-NK cells offer several advantages. One significant benefit is the source of immune cells. While autologous T cells from patients are often limited, especially after pre-treatment, CAR-NK cells can be generated from NK-92 cell lines and induced pluripotent stem cells (iPSCs) [31]. Currently, five clinical trials are utilizing CAR-NK cells derived from NK-92 cell lines [31]. Additionally, CAR-NK cells have been shown to reduce cytokine release syndrome (CRS) and neurotoxicity, and they employ different mechanisms to kill tumor cells compared to CAR-T cells. The CAR construct is similar to the first and second generation of CAR-T, but CAR-NK constructs have been improved with the addition of NK-specific intracellular signaling domains, such as DAP12 and 2B4. Notably, 2B4, a member of the signaling lymphocytic activation molecule (SLAM) family, transduces activation signals through the SLAM-associated protein (SAP), which results in NK cell activation, enhancing cytotoxicity and interferon-gamma production, which improves anti-tumor efficacy compared to 4-1BB-CD3ζ CAR-NK constructs [32]. 

**Figure 3:**
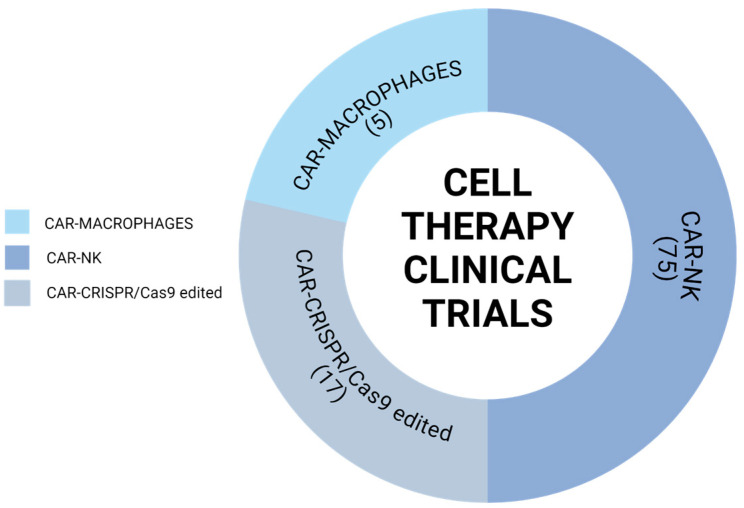
Cell therapy clinical trials as of July 2024 include a variety of approaches, as illustrated by the pie chart displaying data from clinicaltrials.gov. The chart highlights the number of clinical trials involving CAR-M (5 trials), CAR-NK (75 trials), and CAR-CRISPR/Cas9 (17 trials). These trials encompass treatments for both hematological and solid tumors.

The first CAR-NK cell clinical trial (NCT00995137, clinicaltrials.gov) https://clinicaltrials.gov/ (accessed on 27 June 2024) commenced in 2009. Today, there are 75 registered studies on clinicaltrials.gov evaluating the feasibility, safety, and efficacy of CAR-NK cells in cancer treatment [33]. Similar to CAR-T cell therapies, most CAR-NK cell trials target markers on hematopoietic malignancies, such as CD19, CD20, CD22, and BCMA. Notably, there are also CAR-NK cell clinical studies focusing on solid malignancies, including prostate cancer, renal carcinoma, ovarian cancer, and lung cancer, which are typically less responsive to CAR-T cells. These CAR-NK cells target markers such as HER2, NKG2D, mesothelin, and PSMA expressed on these solid tumors (Figure 3, Supplementary Table S2).

A relatively new field is CAR-Macrophages (CAR-M), which researchers have employed to address solid tumors due to their potential for immune cell trafficking and ability to infiltrate the immunosuppressive TME. Macrophages are professional phagocytes and antigen-presenting cells highly specialized in removing aging, injured, dead, and mutated cells or cell debris. Macrophages are essential in innate immunity, maintaining communication between innate and adaptive immunity and playing a vital role in infections and tumorigenesis. One advantage of CAR-M cells is the source of origin; they can be derived from immortalized monocyte cell lines and iPSCs as CAR-NK [34]. The CAR-M construct has the same structure as CAR-T, consisting of an scFv against several targets (e.g., HER2, CD19, and mesothelin), a CD8 hinge and transmembrane domain, and a CD3ζ intracellular domain [31]. However, CAR-M cells have advantages over CAR-T and CAR-NK cells since CAR-M can directly use the CD3ζ intracellular domain to induce phagocytosis signaling, release cytokines, and increase antitumor activity in the TME to better target solid tumors. Since CAR-M therapy is a relatively new field, only five clinical trials are currently targeting both hematological and solid tumors. This limited yet growing area of research highlights the potential of CAR-M cells to address a broader range of cancers (Figure 3, Supplementary Table S3).

Recent studies have demonstrated that combinatorial immunotherapy using CAR-M and CAR-T cells can effectively kill tumor cells in vitro. The inflammatory factors secreted by CAR-T cells increase the expression of costimulatory ligands, such as CD86 and CD80, on CAR-M. This enhances the cytotoxicity of CAR-M by promoting macrophage M1 polarization and may further boost the fitness and activation of CAR-T cells, leading to significantly enhanced cytotoxicity. This study provides the first proof-of-concept that CAR-M can synergize with CAR-T cells to kill tumor cells, introducing a novel combinatorial immunotherapy approach [35]. Additionally, alternative delivery methods for CAR-T have been exploited. While all clinically approved CAR-T therapies currently use the intravenous administration route, recent reports have demonstrated the efficacy of locoregional injection of CAR-T in mouse models of adenocarcinoma and glioblastoma. Encapsulating CAR T cells in a fibrin gel for delivery into the tumor resection cavity has been shown to enhance persistence and functionality, resulting in improved antitumor activity [36,37,38]. Although this promising immunotherapeutic approach has shown optimal results, several challenges remain, particularly concerning mechanisms of resistance against CAR-T cells. These resistance mechanisms can be antigen-dependent, including antigen escape, antigen shedding, antigen heterogeneity, anti-CAR antibodies, or T cell driven, such as CAR-T cell exhaustion and a non-permissive microenvironment [39]. The latest strategy developed to address these challenges involves novel dual CAR-T cells targeting two distinct antigens. This approach aims to reduce general toxicities and combat the development of resistance mechanisms [40].

### 2.1. CRISPR-Based Gene Editing in CAR-T Therapy

Genome editing has revolutionized the field of genetic engineering, providing unprecedented precision and efficiency in modifying cellular genomes. In the context of immunotherapy, particularly for T cells, these advancements hold significant promise for treating a wide array of diseases, including cancer and viral infections. Despite decades of genetic engineering of primary human T cells, the process still requires improvement. The lack of widely accessible tools for efficiently and precisely engineering T cells in a targeted manner has limited their applicability as a living drug [41]. While CAR-T cell therapy has achieved remarkable clinical responses in hematological malignancies, 40–60% of patients eventually relapse after treatment [14]. This underscores the need for further advancements in genetic engineering techniques to enhance the efficacy and durability of CAR-T cell therapies, ensuring they can provide sustained benefits to patients.

CRS is a significant challenge associated with CAR-T cell therapy, often resulting from the release of various interleukins (IL-1, IL-2, IL-4, IL-6, IL-8, IL-10, TNF-α, etc.). While certain interleukins, such as IL-12 and IL-15, enhance anti-cancer activity, and IL-18 activates IFN-γ to further promote CAR-T cell proliferation [42], excessive cytokine release can lead to severe toxicity. Genetic strategies to modulate cytokine signaling during CAR-T cell activation and expansion hold the potential for enhancing antitumor activity, improving T cell persistence, and reducing toxicity.

CRISPR-Cas9 systems have been employed to incorporate transgene knock-in approaches, allowing for precise genetic modifications [43]. These systems, combined with viral or non-viral DNA delivery methods, enable simultaneous bi-allelic or sequential gene targeting to engineer T cells with site-specific expression cassettes [44]. An intriguing example of this approach is provided by Ode et al. [45], where IL-15 was knocked into the IL-13 gene locus, placing it under the control of the endogenous IL-13 promoter, which is highly active upon T cell activation. Furthermore, knocking out genes that drive neurotoxicity and CRS, such as GM-CSF and IL-6 [43], using CRISPR-Cas9 editing, may result in potent and persistent cell therapies. Studies have shown that GM-CSF knockout CAR-T cells maintain normal functions and exhibit increased antitumor activity in vivo, leading to improved overall survival compared to CD19 CAR-T cells [43,46]. Recent advancements have demonstrated that knocking out the GM-CSF gene using CRISPR/Cas9 not only enhances CAR-T cell antitumor activity and survival but also reduces neuroinflammation and CRS [47]. Furthermore, studies have shown that deleting the TGF-beta receptor II in CAR-T cells via CRISPR/Cas9 can increase the population of central and effector memory subsets within circulating CAR-T cells [14], improving their persistence and efficacy.

Another important step in improving CAR-T cell therapy with CRISPR/Cas9 is the potential to reduce CAR-T cell exhaustion [14]. A hallmark of T cell exhaustion is the persistent expression of inhibitory receptors, such as PD-1, CTLA-4, LAG3, and TIM3. These receptors transmit inhibitory signals upon binding to their ligands, leading to T cell exhaustion. PD-1 and CTLA-4, in particular, are pivotal immune checkpoints in this process, with PD-1 being a key marker of T cell exhaustion that functions during the late stages of T cell activation [14]. CRISPR/Cas9 can be utilized to mitigate exhaustion and maintain the CAR-T cell effector function and persistence. Two main approaches are proposed: eliminating immune checkpoints and disrupting specific regulatory factors associated with exhaustion. The first approach involves using CRISPR/Cas9 to delete immune checkpoints such as CTLA-4 [48]. Studies have shown that this can not only enhance the efficacy of CAR-T cell therapy but also reduce exhaustion, thereby improving overall therapeutic outcomes. The second approach involves the use of CRISPR/Cas9 to knock out exhaustion-related genes such as ID3 and SOX4 [49]. Research has demonstrated that this can delay CAR-T cell exhaustion and enhance cytotoxic activity. Additionally, disrupting PTP1B with CRISPR/Cas9 can inhibit cytokine-induced JAK/STAT signaling activation, leading to improved CAR-T cell activity in solid tumors [50]. Moreover, CRISPR/Cas9-mediated knockout of A2AR enables CAR-T cells to resist adenosine-mediated immunosuppression [51].

One of the emerging uses of CRISPR/Cas9 is its application in screening for new targets for long persistence, markers, and targets for exhaustion and memory phenotype in order to globally ameliorate CAR-T therapy. Indeed, the current studies mostly focus on knocking out genes that proved to have a crucial role in regulating CAR-T cell differentiation, survival, and effector functions, ultimately leading to an enhancement of CAR-T cell clinical efficacy [14]. This strategy utilizes CRISPR/Cas9 gene editing and libraries of sgRNAs capable of targeting every gene in the genome [14]. Examples of this application are the works performed by Jain et al., in which they identified RASA2 as a novel target to boost both the persistence and effector functions of CAR-Ts in different genome-wide CRISPR KOs under several immunosuppressive models [52]. Other important works identified MED12 as a regulator of CAR-T cell effector activity on CRISPR KOs [53], BATF depletion as an improver of CAR-T cell resistance to exhaustion [54], and the pharmacological inhibition of p38 kinase as an enhancer of CAR-T cell persistence and anti-tumor efficacy [55].

Ultimately, CRISPR/Cas9 could be a pivotal tool in reducing the manufacturing costs of CAR-T cell therapies. The clinical development of these therapies is frequently hindered by the low yield and poor functionality of mature, autologous peripheral blood T cells obtained from elderly and heavily pretreated patients [43]. A potential solution involves sourcing healthy donor leukocytes to create ‘universal’ T cells with enhanced in vivo persistence and antitumor efficacy. However, producing off-the-shelf CAR-T cell products presents challenges because it requires multiple genome edits in a limited number of differentiated T cells to prevent alloreactivity and immunogenicity while enhancing robust, tumor-specific activity [43].

Several clinical trials are also investigating the safety and efficacy of CRISPR/Cas9 technology to reduce CAR-T cell toxicity. Notable trials include NCT06128044, NCT03545815, and NCT03545815. Most trials are in Phase I, aiming to evaluate the safety and efficiency of universal CAR-T cells in hematological malignancies. Additionally, there are trials focused on immune checkpoint-disrupted CAR-T cells, with some producing encouraging results. The outcomes of these clinical trials demonstrate a promising future for CRISPR-engineered CAR-T cell therapy in clinical applications, improving the efficiency, stability, and safety of CAR-T cells (Figure 3, Supplementary Table S4).

These findings underscore the potential of CRISPR/Cas9-mediated gene editing to optimize CAR-T cell therapies, making them more effective and safer for clinical use.

### 2.2. Engineering Nanoparticles for Effective In Vivo CAR-T Therapy

In recent decades, scientific research has made tremendous progress in enhancing adoptive therapies for generating CAR-T cells to treat hematologic and solid tumors. Indeed, innovative CAR designs and long-term clinical trial results have emerged, solidifying the therapeutic potential of this synthetic immune receptor [56]. However, this success presents significant challenges to overcome, such as (i) time-dependent good manufacturing product (GMP) production, (ii) scaling up product, (iii) reducing T cell patient lymphodepletion to generate CAR-T cell, and (iv) a bespoke process of ex vivo CAR-T cell manufacturing to broadly apply this therapy across various cancer indications [57].

Currently, LNPs represent the most clinically advanced nucleic acid delivery system, following their successful use in multiple mRNA COVID-19 vaccines [58]. The success of this strategy is attributable to the biochemical properties of the lipid shell, which protect and stabilize the mRNA molecule from degradation. The LNP mixture primarily comprises ionizable cationic lipids, helper lipids such as phospholipids and cholesterol, and polyethylene glycol (PEG)-conjugated lipids [58]. These components collectively facilitate physiological endocytosis, endosomal escape, and the release of the mRNA cargo into the cytosol for translation [58]. Interestingly, LNPs may serve as a robust platform to encapsulate mRNA encoding the CAR gene for direct in vivo transfection of T cells. One strategy to target T cells directly is to modify the lipid shell. Similar to other nanoparticle types, the LNP strategy involves conjugating an antibody specific to T cells, thereby enhancing the engineering of these cells.

In the field of anti-tumor therapies, nanoparticles have been utilized in diverse applications, including vaccination against neoantigens in clinical trials [59]. Of note, nanoparticles have further demonstrated their versatility in the delivery of mRNA/DNA encoded CAR-T and TCR engineering T cells in vivo using lymphocyte markers like CD3 and CD4 as a promising strategy to enhance T cell specificity [60,61,62,63,64,65]. This technology may offer several potential advantages, including rapid treatment access for aggressive diseases, improved safety profiles, and potentially lower costs [58]. From a biological perspective, these benefits stem from the transient expression of the desired construct. This transient expression reduces the risk of severe toxicities like CRS and immune effector cell-associated neurotoxicity syndrome (ICANS), while also minimizing off-target effects [66].

Among emerging technologies, polymer-, lipid-based nanoparticles are showing great promise for CAR-T cell development due to their potential for safe and cost-effective T cell engineering. Engineered viral vectors, such as enveloped delivery vehicles (EDV) derived retrovirus-like particles (VLPs), have also garnered significant attention. These technologies represent a novel approach for engineering T cells in vivo using Cas9 ribonucleoprotein (RNP) [65]. Polymer-based nanoparticles, in particular, rely on the interaction between cationic molecules (positive charge) and the negative charge of the phosphate groups in nucleic acids, leading to the formation of structures called poly-complexes [67]. Various polymers have been identified and characterized for their chemical and biological properties, making them a viable platform for protecting nucleic acids from degradation and efficiently delivering them into target T cells in vivo [68]. 

For T cell transfection engineering, several cationic polymers, including polyethyleneimine (PEI), poly(2-dimethylaminoethyl methacrylate) (PDMAEMA), and poly(β-amino esters) (PβAE), have demonstrated promising results. These approaches have shown potential in targeting CD19-positive leukemia in mouse models [69]. Smith et al. pioneered the delivery of a CAR plasmid construct to target and treat leukemia using 194-1BBz CAR-encoding transgenes. Their research focused on polymeric nanoparticles, specifically PβAE-based nanosystems, to deliver the CAR construct directly into circulating T cells, enabling the expression of leukemia-specific CARs. The formulation was further improved by combining a polymer nanocarrier with an anti-CD3 antibody, which allowed for selective binding to T lymphocytes in vivo with minimal off-target effects. This approach demonstrated antitumor activity in a mouse model of B-cell acute lymphoblastic leukemia [60]. 

Additionally, the researchers tested this therapeutic approach in a solid tumor model, evaluating its potential in a prostate cancer model using mRNA nanocarriers encoding prostate-specific CARs. Their findings showed that tumor-specific CARs could improve the survival of mice with established disease [61].

The in vivo targeting of CD3 and CD7 antibody-conjugated lipid nanoparticles (Ab-LNPs) has successfully delivered CAR mRNA, resulting in significant CAR-T cell populations capable of depleting B cells and releasing cytokines in a controlled, dose-dependent manner. Notably, the CD3-LNPs demonstrated superior performance, achieving high transfection rates and potent CAR expression. Moreover, the transient approach allowed for repeated dosing, thereby reducing long-term side effects, such as cytokine release syndrome (CRS), associated with permanent CAR expression [63].

Zhou et al. introduced an LNP system modified with a CD3 antibody and loaded with a plasmid containing IL-6 shRNA and CD19-CAR genes (AntiCD3-LNP/CAR19 + shIL6). These nanoparticles target T cells, transfecting them to become CAR-T cells that knock down IL-6, thereby reducing CRS and effectively targeting CD19-expressing leukemia cells. In vivo experiments demonstrated that these nanoparticles could stably transfect T cells and significantly prolong the survival of leukemia model mice. Furthermore, the IL-6 knockdown mitigates CRS by reducing pro-inflammatory cytokines such as IL-2, TNF-α, and IFN-γ after T cell reprogramming, thus improving the safety of CAR-T treatments [62].

A key feature of CAR-T therapy is the regulation of immune checkpoints, which can facilitate tumor escape. By incorporating a regulatory element into the LNPs, the in vivo CAR-T cell engineering platform was enhanced with immune checkpoint inhibition. The LNPs, co-encapsulating CAR mRNA and siRNA targeting PD-1, successfully achieved potent, transient CAR expression and temporary T cells PD-1 inhibition [64]. As opposed to the antibody-conjugated strategy, Álvarez-Benedicto et al. investigated the ability of a modified LNP formulation to reach the target organ without the addition of T cell-recognizing antibodies in a B cell lymphoma model. They modified the traditional four-component LNP delivering an mRNA construct for CD19-41BB by incorporating 10% 18:1 PA(1,2-dioleoyl-sn-glycero-3-phosphate) into the canonical lipids (5A2-SC8, DOPE, cholesterol, and PEG-DMG) [58], achieving an antitumor effect [70].

Hamilton et al. explored a novel approach for delivering genome editing tools to specific cells using Cas9-packaging enveloped delivery vehicles (Cas9-EDVs). The study focused on overcoming the limitations of conventional vector viruses, which lack cell-type selectivity. The researchers employed antibody fragments on membrane-derived particles to target specific cell surface markers, thereby achieving selective delivery of CRISPR-Cas9 components to the desired cells both ex vivo and in vivo [65]. The key innovation lies in using multiplexed targeting molecules represented by CD3 and CD4 receptors that enable precise delivery of genome editing tools to human T cells, facilitating the generation of genome-edited CAR-T cells in humanized mice. Moreover, this method allowed targeted editing without affecting bystander cells, thus minimizing off-target effects and showcasing their potential for therapeutic applications. Overall, the study highlighted the programmability and versatility of Cas9-EDVs in achieving optimized receptor-mediated delivery and genome editing in various cell types and in vivo [65].

**Figure 4:**
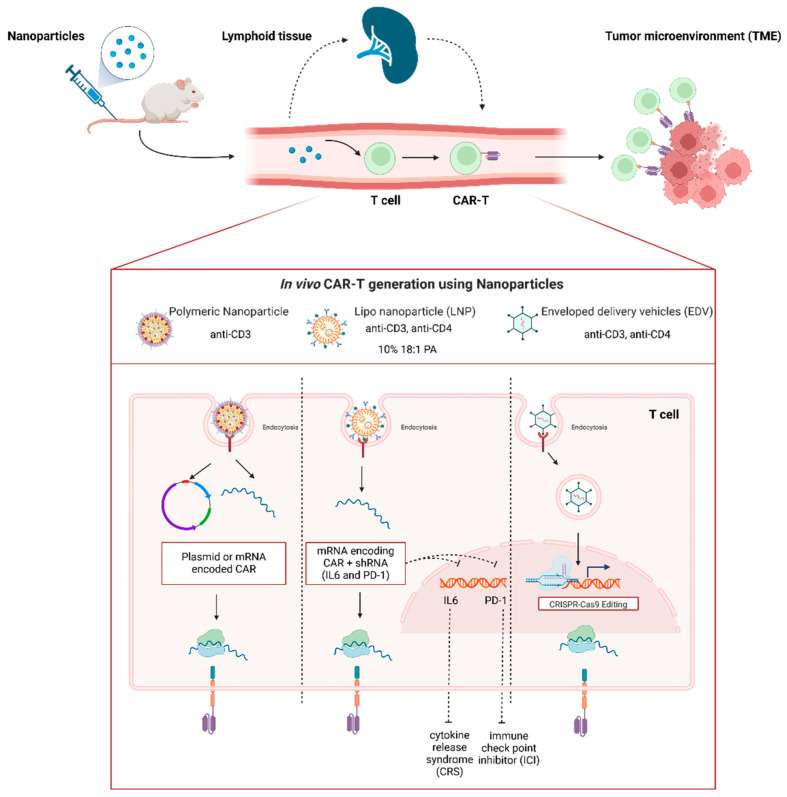
Engineered nanoparticles for in vivo CAR-T cell production. The top section of the figure illustrates the schematic process of in vivo CAR-T cell generation through the administration of nanoparticles, highlighting their anti-tumor efficacy. The bottom section depicts the diverse types of nanoparticles, and the strategies utilized in preclinical models.

These findings have empowered new opportunities in cancer nanomedicine to prove a valid strategy to treat tumors in vivo. However, to maximize its impact, we need to address limitations like optimizing antibody conjugation and preventing CAR-T cell off-target effects (Figure 4).

### 2.3. Role of Extracellular Vesicles in Immunotherapy

EVs have been identified as crucial components in cell-to-cell signaling, as well as structures involved in the elimination of cellular waste. Their role has been extensively studied in the interaction between tumors and immune cells, revealing significant insights into their function [71].

EVs play a pivotal role in T cell development. Thymic epithelial cell-derived EVs transport tissue-restricted antigens (Ag) to conventional DCs, contributing to the negative selection of T cells that are directed against self-antigens [72]. Additionally, EVs can enhance DC function by “cross-dressing”, wherein EVs concentrate on the DC surface, thereby promoting the formation of immune synapses and T cell activation. Moreover, EVs are capable of delivering peptide-major histocompatibility complexes (pMHC) as well as intact antigens directly to APCs, which can internalize and process these antigens for indirect presentation [73]. This cross-presentation mechanism is particularly significant when tumor-associated antigens are processed and presented on MHC class I molecules, thereby priming naïve CD8+ T cells. Furthermore, this mechanism is instrumental in developing immunity against viruses, enhancing the immune response induced by vaccination and tolerance induction. By facilitating these processes, EVs contribute significantly to both immune surveillance and the potential development of therapeutic strategies targeting cancer and infectious diseases.

Marcoux and colleagues demonstrated that microvesicles, medium-sized vesicles derived from platelets, function effectively as units for antigen presentation. These microvesicles deliver pMHC class I complexes and co-stimulatory molecules, such as CD40L, CD40, and OX40L, on their surface. Additionally, they carry functional 20S proteasomes, which generate peptides for antigen presentation, subsequently activating CD8+ T cells [74]. Due to these features, EVs can be utilized as vaccine formulations. Dendritic cell-derived exosomes (DC-Ex) deliver numerous immunoregulatory molecules, providing a stable platform with high safety, easy preparation, and preservation while enhancing immune response specificity [75]. DC-Ex loaded with patient-derived tumor antigens have been shown to home in on lymph nodes and trigger T and B cell immune responses in mouse models of melanoma and colon cancer [76]. This system inhibits tumor growth by stimulating the release of various factors and promoting T cell infiltration at the tumor site. Concurrently, activated B cells secrete antigen-specific antibodies, leading to a synergistic effect of humoral and cellular immunity [76]. These findings highlight the potential of EVs, particularly DC-Ex, as versatile and effective components in cancer immunotherapy, offering promising avenues for enhancing anti-tumor immune responses through both cellular and humoral mechanisms.

In recent years, other platforms, such as engineered EVs and biomimetics, have been developed to trigger immune responses against cancer. For example, T cells transfected with GFP-PD-1 lentivirus produce EVs that express PD-1 and neutralize PD-L1, thereby enhancing the killing activity of TILs in melanoma [77]. Another system utilizes HELA EVs enriched with TLR3 agonists and immunogenic cell death inducers to activate DCs in situ in a breast cancer model [78]. Recently, Liu and colleagues developed an engineered DC-derived exosomal platform using biomimetic synthesis technology [79]. This Antigen Self-Presentation and Immunosuppression Reversal (ASPIRE) technology employs DC membranes to deliver CD80/CD86 costimulatory molecules along with anti-PD-1 antibodies. This approach reverses tumor-induced suppression and promotes T cell reactivation in a model of Lewis lung carcinoma [79]. These innovative platforms represent significant advancements in cancer immunotherapy, offering new strategies to enhance the immune system’s ability to target and eliminate tumor cells.

Following the CAR-T revolution, researchers have increasingly focused on the use of CAR-T-derived EVs. Traditional T cell therapy faces challenges in solid tumors due to poor penetration within the tumor mass. However, EVs can efficiently cross biological barriers and penetrate tumors. Studies have shown that EVs expressing specific CARs on their surface can kill tumor cells by delivering granzyme A and perforins [80]. Additionally, EVs do not express PD-1 on their surface, making them particularly effective when used in conjunction with PD-L1 therapy [80]. A similar system was proposed by Cheng, wherein Expi293F cell-derived exosomes were armed with monoclonal antibodies specific for human T cell CD3 and epidermal growth factor receptor (EGFR), along with immune checkpoint modulators, PD-1 and OX40 ligand (OX40L). This genetically engineered multifunctional immune-modulating exosomes (GEMINI-Exos) platform activated T cells against EGFR-positive triple-negative breast cancer and enhanced anti-tumor immunity in mice [81].

Recently, a type of “smart exosomes” was developed and functionalized with CD62L (L-selectin, a gene for lymphocyte homing to lymph nodes) and OX40L (CD134L, a gene for effector T cell expansion and regulatory T cell inhibition) by increasing its expression in parental cells. Compared with control exosomes, CD62L/OX40L-enriched exosomes displayed a strong homing capacity to tumor-draining lymph nodes [82]. Moreover, the injection of these exosomes activated effector T cells and inhibited Treg induction, thereby amplifying the antitumor immune response and inhibiting tumor development.

These advancements in EV technology represent significant progress in enhancing the efficacy of cancer immunotherapy, particularly for solid tumors, by overcoming the limitations of traditional T cell therapies and improving tumor targeting and immune activation.

### 2.4. Vaccines

Vaccines were initially developed to prevent infectious diseases, but recently, the concept of using vaccines to treat established cancers has emerged as a significant challenge. Although immunotherapy, particularly with the development of CAR-T cell therapy, has shown great efficacy, its success depends on identifying specific antigens. Vaccines can enhance efficacy by targeting a broader range of intracellular antigens. Furthermore, while checkpoint inhibitors target only specific subsets of “inflamed” cancer cells, vaccines can prime tumor-reactive T cells through TCR signaling, potentially improving the overall therapeutic outcome.

Antitumor vaccines can be classified based on the targeted antigen, which may include whole tumors, tumor cells, proteins, peptides (long or short), and RNA or DNA (delivered directly or via viral vectors). They can also be categorized by their delivery systems, such as carrier proteins, cells (e.g., DCs), proteins (e.g., CD40 ligand (CD40L)), or chemicals (e.g., oil–water emulsions and Toll-like receptor (TLR) agonists) [83]. Most tumor-associated antigens (TAA) are expressed in the cytoplasm and are not accessible by standard therapies like monoclonal antibodies, CAR-T cells, or bispecific T cell engagers. However, T cells can detect TAA when they are presented with HLA on tumor cells, although the absence of costimulatory molecules often leads to T cell anergy or exhaustion. Dendritic cells play a crucial role in TAA presentation, and specific subsets, particularly type 1 conventional DCs, have been shown to be important in cross-presentation [84].

The first tumor vaccines developed in the 1990s used lethally irradiated tumor cells combined with GM-CSF. This approach blocked tumor cell replication through irradiation and stimulated the recruitment of DCs to the injection site with GM-CSF, promoting DC survival, maturation, and the homing of antigen-loaded DCs to lymph nodes [85]. For instance, in one of the initial studies, 18 patients with relapsed, measurable indolent non-Hodgkin lymphoma were vaccinated with DCs loaded with killed autologous tumor cells. One-third of the patients exhibited a complete response, while the rest maintained stable disease, with only four experiencing disease progression. The clinical response was associated with a reduction in regulatory T cells and an increase in natural killer cells and effector memory T cells [86]. Recently, a Phase I study demonstrated that loading DCs with pancreatic ductal adenocarcinoma lysate induced a response, as indicated by increased activated PD-1+ circulating T cells [87]. However, this system showed limitations and low efficacy in two Phase III clinical trials [88].

An ex vivo DC antigen-loading method was subsequently developed. This process begins with apheresis, followed by the in vitro generation of DC vaccines through the differentiation of monocytes into immature DCs. Tumor antigens are then loaded during the final activation phase of mature DCs. This approach enables the generation of DCs that not only present tumor antigens but also express costimulatory molecules and secrete cytokines [89]. Some studies have demonstrated enhanced DC activity using a combination of cytokine cocktails and Toll-like receptor agonists [90]. This ex vivo loaded DC method circumvents the issue of endogenous DC dysfunction often observed in tumor patients. However, the production process is labor-intensive and costly. Additionally, the short stability of antigen presentation and insufficient priming of T cells pose significant challenges to the effectiveness of this vaccine type.

With advances in sequencing and immunogenic neoantigen screening techniques, DC loading has been improved by using patient-specific antigen mRNA transfection [90]. A Phase 2 study investigated the effects of vaccinating 30 patients with Acute Myeloid Leukemia and at very high risk of relapse, using DCs electroporated with Wilms’ tumor 1 (WT1) mRNA as a post-remission treatment. The researchers observed an antileukemic response in 43% of patients, with the long-term clinical response correlating with increased circulating frequencies of polyepitope WT1-specific CD8+ T cells [91]. In recent years, many peptide-based vaccines have been developed due to their ease of design and production. This system utilizes peptides that encode a portion of a known tumor antigen, which can be taken up by antigen-presenting cells (APCs) to activate a T cell response [92]. Most of these peptides are administered with adjuvants that enable APC activation, such as oil emulsions or double-stranded oligonucleotides like poly-ICLC [93]. Early trials of peptide-based vaccines focused on administering major histocompatibility complex (MHC) class I-restricted 8–9 amino acid peptides, which form peptide–MHC complexes for T cell activation without needing uptake and processing by APCs. Recently, research has shifted from short to long peptide designs to avoid nonspecific presentation by non-APCs, which can lead to T cell anergy [94]. For example, the synthetic long peptide vaccine ISA101 induced T cell responses and tumor regression in the majority of patients with vulvar intraepithelial neoplasia. Additionally, a study demonstrated that combining ISA101 with anti-PD-1 therapy increased clinical responses more than either therapy alone, even in PD-L1-negative tumors [95].

Another category of cancer vaccines is DNA-based vaccines. These typically involve plasmids encoding tumor antigens, which are taken up by cells and expressed as specific proteins. This mechanism is more efficient and stable because the injection of foreign genetic material is inherently immunogenic [96]. However, the need for intracellular delivery and expression of DNA has posed a barrier to the efficacy of these vaccines. Recently, various strategies have been developed to enhance DNA delivery into APCs. One approach, called GeneGun, uses heavy metal nanoparticles coated with DNA to forcibly introduce the molecules into immature DCs, which then process and present them in lymph nodes, where T cells are activated [97]. Another method employs electroporation to increase DNA uptake by muscle cells, which has been shown to generate a long-lasting expression of the transfected DNA plasmid [98,99]. This technique results in a thousand-fold increase in antigen delivery compared to naked DNA injection alone [100]. Additionally, electroporation induces a local site of inflammation and cytokine release, recruiting DCs and macrophages [101]. 

Recently, researchers proposed a novel approach for using vaccines. Instead of targeting cancer cells directly, the vaccines are designed to enhance CAR-T cell efficacy in the fight against solid tumors. Ma et al. demonstrated that in vivo vaccine boosting of CAR-T cells engages the endogenous immune system to circumvent antigen-negative tumor escape. Vaccine-boosted CAR-T cells showed a metabolic shift toward oxidative phosphorylation (OXPHOS) of IFN-γ produced by CAR-T cells. This promoted antigen spreading and the engagement of endogenous T cells. Thus, vaccine boosting can be a clinically translatable strategy to improve responses against solid tumors [102].

## 3. Conclusions and Future Directions

Despite significant successes in treating hematological malignancies, addressing the challenges posed by solid tumors requires innovative solutions. The intricate interplay between the tumor microenvironment and immune system presents considerable challenges, but advancing our understanding of these complexities is vital for overcoming tumor evasion mechanisms and mitigating the side effects associated with CAR-T cell therapy.

Strategies such as locoregional delivery methods, the development of armored CAR-T cells, and the engineering of other immune cells like NK cells and macrophages, which can secrete cytokines or undergo multiplex gene edits, are paving the way for more effective treatments. These advancements aim not only to enhance the cancer-killing capabilities of immune cells within solid tumors but also to reduce systemic toxicities, which have previously hindered the scalability of such treatments.

CRISPR/Cas9 technology offers substantial benefits in reducing CAR-T cell manufacturing costs through multiple mechanisms. It improves the efficiency and success rate of genetic modifications, minimizes waste, and enables the production of “universal” CAR-T cells from healthy donors, thus eliminating the need for patient-specific manufacturing. This advancement not only cuts costs and production time but also enhances the yield and functionality of CAR-T cells by modifying genes to improve T cell survival and proliferation. Furthermore, CRISPR/Cas9 reduces alloreactivity and immunogenicity, facilitating the creation of universally applicable CAR-T cells and simplifying the treatment process. By optimizing CAR-T cells for better in vivo persistence and antitumor activity, CRISPR/Cas9 further decreases the required therapeutic doses, thereby lowering production costs.

In addition, the use of vaccines, LNPs, and EVs represents a groundbreaking advancement in medical science, offering innovative and effective strategies for disease prevention and treatment.

Looking ahead, the continued integration of synthetic biology, gene editing, and innovative delivery methods, will refine cell therapies, potentially establishing them as the cornerstone of cancer treatment, even in the most challenging solid tumor settings. The rapid pace of discovery and translation in this field heralds a new era in oncology, where the full potential of CAR-T cells, NK cells, macrophages, engineering nanoparticles, vaccines, and EVs can be harnessed to combat all cancers with unprecedented precision.

## Data Availability

Data are contained within the article.

## Supplementary Materials

The following supporting information can be downloaded at: https://www.mdpi.com/article/10.3390/medsci12030043/s1, Table S1: FDA approved CAR-T Trials; Table S2: CAR-NK Clinical Trials; Table S3: CAR-M Clinical Trials; Table S4: CRISPR/Cas9 Clinical Trials.
